# The key values and factors identified by older adults to promote physical activity and reduce sedentary behaviour using co-production approaches: a scoping review

**DOI:** 10.1186/s12877-023-04005-x

**Published:** 2023-06-16

**Authors:** Elysa Ioannou, Henglien Lisa Chen, Vicky Bromley, Sam Fosker, Khalid Ali, Avanka Fernando, Ekow Mensah, Sally Fowler-Davis

**Affiliations:** 1 Sport and Physical Activity Research Centre (SPARC), Sheffield Hallam University, Sheffield, UK; 2 University of Sussex (Social Work and Social Care), Brighton and Hove, UK; 3 Royal London Hospital, London; Founder, Cush Health, London, UK; 4 Brighton and Sussex Medical School, Brighton and Hove, UK; 5 University Hospitals NHS Trust, Nottingham, UK; 6 Advanced Wellbeing Research Centre (AWRC), Sheffield Hallam University, Sheffield, UK

**Keywords:** Scoping review, Older adults, Physical activity, Co-production

## Abstract

**Background:**

Inactivity and sedentary behaviour in older adults adversely impacts physical function, reduces social networks, and could contribute to population healthcare costs. To encourage and support the planning and uptake of physical activity by older adults, it is important to understand what physical activity means to older adults. Therefore, the aim of this scoping review was to collate what older adults have self-identified as the key factors for sustaining and increasing their physical activities.

**Methods:**

Arksey and O’Malley’s Scoping Review framework was used to guide the review process. SCOPUS, ASSIA, PsychINFO and MEDLINE databases were searched. Studies were eligible for inclusion if they were peer-reviewed, the target population were older adults (aged 55 and above), co-production related research approaches were explicitly stated in the methods and there was a focus on design of physical activity interventions or products to support or enhance physical activity. Assets and values important for physical activity were first extracted from included studies and were subsequently thematically analysed. Themes are presented to provide an overview of the literature synthesis.

**Results:**

Sixteen papers were included in the analysis. Data from these papers were gathered via designing interventions or services (*n* = 8), products (*n* = 2), ‘exergames’ (*n* = 2) or mobile applications (*n* = 4). Outcomes were varied but common themes emerged across papers. Overarching themes identified by older adults were associated with a desire to increase activity when it was accessible, motivational, and safe. In addition, older adults want to enjoy their activities, want independence and representation, want to stay connected with families and friends, be outdoors, familiarity, activities to be tailored and resulting in measurable/observed progress.

**Conclusions:**

Population demographics, personal attributes, and life experiences all affect preferences for physical activity. However, the key factors identified by older adults for increasing physical activity were common—even in separate co-production contexts. To promote physical activities in older adults, activities must fundamentally feel safe, provide a sense of social connectedness, be enjoyable and be accessible in terms of cost and ability.

**Supplementary Information:**

The online version contains supplementary material available at 10.1186/s12877-023-04005-x.

## Background

The Mid 2020 census estimates that just over 12.5 million people in the UK are aged 65 and over [1] and projected assumed improvements in mortality mean that current life expectancy at birth in the UK in 2020 is 87.3 years for males and 90.2 years for females [2]. A large body of research has demonstrated the potential adverse impact of sedentary behaviour on individuals, communities and societies, such as the decline of physical functions [3–5], reduced social networks [6] and the impact on healthcare costs [7, 8].

Improving PA enhances quality of life for older adults and is a key policy and research concern [9, 10]. Positive gerontology and innovation programs consider the concept of ‘active ageing’ as important to this end. The World Health Organization (WHO) adopted the term ‘active ageing’ [11] as a driving concept in their report: *A Global Strategy for Healthy Ageing* [12]*.* Now a prevailing concept in policy and research, active aging is defined as “the process of developing and maintaining the functional ability that enables wellbeing in older age” [13]. Active Ageing is chiefly concerned with the promotion of PA, which has been defined as “any bodily movement produced by skeletal muscles that results in energy expenditure” [14]. Arguably, a more holistic understanding of PA is welcomed, rather than limiting PA to ‘exercise’ which could constitute the disengagement and alienation of PA among older adults. Increased PA is widely recognised both nationally and internationally as beneficial for improving quality of life in older adults [9, 10, 13–15]. Studies show that the adverse effects of ageing can be mitigated by regular exercise [3] and that cognitive decline and dementia can be slowed [16–18]. Therefore, promotion of PA in older adults is essential and important in care policy and practice.

Despite raising awareness of the importance of PA amongst the public, PA continues to decrease over the life course and as people age [19]. Recent studies highlight that interventions aimed at increasing PA amongst older adults has not affected a great change in behaviour [20, 21]. Crombi et al*.,* (2004) found older adults expressed that a primary barrier preventing them from exercising is simply a lack of interest [22]. Nonetheless, the reasons for inactivity in older adults are complex. For example, a fear of falling is one of the main reasons that may prevent many older adults from taking part in exercise programmes, especially when living alone. Avoidance of overall activity due to the fear of falling can exacerbate social isolation with risk of reduced life satisfaction [23]. To complicate the situation further, evidence shows that there is resistance to the uptake of and adherence to exercise in the home setting [24].

With this cyclical problem at the forefront, and whilst many previous studies have focused upon improving PA as it pertains specifically to exercise (see for example, Di Loito’s meta-analysis, 2021 [25]), recent policy is leaning towards concentration on improving all forms of PA. The UK government’s latest PA guidelines from the Chief Medical Officer (2019) acknowledge that in sedentary older adults, the health benefits of even small amounts of PA, carried out as part of the daily routine (e.g., carrying shopping or wheeling a wheelchair) could be effective behavioural goals to be acknowledged and encouraged [26]. The World Health Organization (2018:46) asked member states to prioritise (i) the reduction of overall level of physical inactivity and (ii) to reduce within-country disparity in inactivity. The emphasis, placed on the *reduction of inactivity* adds weight to the policy’s departure from formal exercise as a sole solution. Similarly, in July 2019, the UK government launched a green paper, *Advancing our Health: Prevention in the 2020s* to promote active lifestyles by encouraging people to switch from driving to public transport, cycling and walking [27]. This policy focus has moved to increasing PA and away from more structured exercise programmes. Atypical and innovative methods of increasing PA in ways that are both safe and reflective of older adults’ wishes are desirable in moving forward to meet the needs of older adults.

Intervention strategies may have not considered closely enough the individual dispositions, aspirations and biographies that form the foundation of PA preferences and patterns, even when evidence based [28, 29]. While the feasibility of these interventions was evaluated and was good, designing interventions in the first place should consider barriers to engagement. Additionally, to encourage the uptake of PA, we should begin by first gaining some understanding of what PA means to older adults, and it is important to include their voices when planning PA with them. Co-production definitions are diverse across and within disciplinary settings, therefore as highlighted and in agreement with Smith et al*.,* the present review will define co-production and co-produced settings as any typology of research approaches that enable older adults as equal partners in research and designing services, products and interventions in PA [30]. Therefore, the focus of the present paper are the attitudes, barriers and facilitator to PA as captured through the voices of older people in these co-production settings.

### Research questions and aims

This scoping review aims to provide an overview of the range and type of research existing in the co-production space that engaged older adults and included an element that could help to understand what motivates the uptake and maintenance of PA in older adults.

The research questions were as follows:

1. What are the important values (inc. meaning) for increasing activity identified by older adults in the context of co-produced physical activities?
2. What are the important factors (inc. barriers and facilitators) for increasing (or restrict) activity identified by older adults in the context of co-produced physical activities?

## Methods

Arksey and O’Malley’s Scoping Review framework [31] was used to guide the review process.

### Eligibility criteria

A summary of the eligibility criteria is presented in Table 1. To be relevant for the review, studies had to be peer-reviewed. Studies could be international, however, had to be published or accessible in English. The present scoping review aimed to uncover fresh perspectives for recent studies not included in other scoping reviews, therefore, to be included, studies had to be published after 2016. Additionally, the present scoping review took the definition of older adults to mean anyone over the age of 55, therefore, to be included, studies had to included participants aged 55 or over.

**Table 1:** Eligibility of studies

	To be included:	Excluded if:
Type of article	Empirical research that has been peer reviewed	Protocols, theses, editorials and discursive papers and non-peer reviewed articles
Limiters	Studies had to be in English Language and published after 2016	Not published or accessible in English language and published before 2016
Population	Participants had to be older adults (aged 55 +)	Including adults aged < 55 years
Study design	An explicit statement of use of participatory methods and or co-production methods with older adults	Did not use or did not clearly state use of co-production or participatory methods
Outcome	Clear mention of and collection of PA meaning and preferences for/by older adults	Not PA related e.g., sole aim of enhancing overall wellbeing, reduce social isolation, housing or improve access to the environment (“smart cities”)

In terms of study design, to be included, studies had to clearly demonstrate use of any co-production methodology. There are three terms that are often used interchangeably, co-design, co-creation and co-production. For this scoping review, “co-production” is adopted as an overarching term to encapsulate each type of the differing approach together. The present review will focus on PA outputs within differing and diverse co-produced settings. A sister paper is currently being prepared which evaluates the co-production approaches used. This sister paper is also an extensive body of work and addresses discussions on the ‘trueness’ of these co-production settings [32, 33], highlighting the types of approaches used and how they were implemented in each setting.

This was necessary to answer the research question, to aid an understanding of identified values and key factors for PA identified by older adults in a co-production setting. Therefore, studies also had to explicitly state and report older adults’ perceptions and preferences toward PA as part of the co-production process. However, studies were still included if PA was a secondary aim, but PA and outcomes were still a focus of the study and was reported on. Studies were excluded where there was no specific aim to increase or sustain PA. As an example, this could be where the focus was designing “smart cities” or increasing social inclusion. These studies and others were similarly excluded due to an insufficient focus on PA elements.

### Information sources and Search strategy

Four separate databases; SCOPUS, ASSIA, PsychINFO and MEDLINE were searched to extract key health and social care literature. Search terms were developed iteratively. Various combinations of database specific subject headings and keywords were therefore tested for accuracy. The final search strategy was in the combination of Co-creation OR co-design OR co-production AND older adults AND PA OR mobility. These were used in searches of titles and abstracts.

### Selection and Data collection process

The present review was conducted as a precursor to the Zinc Catalyst Project (Grant Number G0606-56). The aim was to inform a co-production activity in a sheltered accommodation. Upon reviewing the papers, it was recognised the scale of data present for analysis. Therefore, the splitting of the reviews into 2 separate papers for publication was necessitated. One, focused on the co-production and methods employed and the present review focused on the specific PA outcomes, assets, and values.

The scoping study was undertaken and the results relating to co-production methods and effectiveness published elsewhere (see Chen et al*.,* 2022 (forthcoming)). The literature was extensive enabling a separate focus on PA outcomes in this paper.

### Synthesis methods

**Fig. 1:**
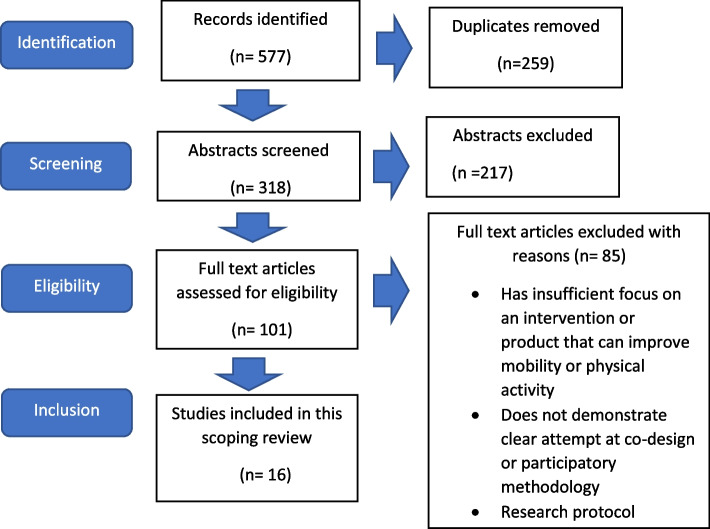
PRISMA flow chart of study selection

Once data was extracted, a table of study characteristics was created. This allowed for the papers’ aims, approach to data collection and outcomes to be summarised clearly. Each paper was assessed for an essential component: that they improved and sustain levels of activity in older adults. The Prisma table below (Fig. 1) corresponds with the selection of studies for PA and for co-production (based on a single study with separate analysis and outcomes (see above). These were summarised and extracted into a table displaying the analogous identified values and factors from each included paper.

Once factors and values associated with PA were identified and extracted from each paper, they were then thematically analysed using a reflexive approach in NVivo 12 to code the data and then identify the main themes. A reflexive thematic analysis facilitated the data-driven approach employed [34]. The lead author (EI) first developed the main themes, and then reflected on these with SFD to further develop these themes following an essentialist view with an experiential orientation in a mainly inductive approach which began with open-coding of data [35]. Evaluation of the data continued iteratively until a final list of themes and their interrelationships were identified. To make sense of the initial free-coding, mind maps were created. These mind maps are displayed in the results. The subsequent main themes were also reported in the findings, including in a table indicating in which papers each theme appeared.

## Results

The initial search yielded 577 records, leaving 318 abstracts for screening once duplicates were removed. One-hundred-and -one full texts of these abstracts were further assessed for eligibility. In the final stage of screening, articles were excluded for one of 3 reasons; due to an insufficient focus on an intervention or product to improve mobility or activity, not demonstrating a clear co-design or participatory methodologies or due to the research protocol. Therefore, the present scoping review included a total of 16 papers.

### Characteristics of included studies

A summary of characteristics of included studies is presented in (Table 2). The 16 papers selected included gathered data on older adults’ preferences for PA via designing interventions or services (*n* = 8), products (*n* = 2), ‘exergames’ (*n* = 2) or mobile applications (*n* = 4).

**Table 2:** Summary of study characteristics

Author	Date	Aim	Country	Approach	PA Outcome
**Design of Interventions / Services**					
Gine-Garriga et al. [36]	2019	Co-create the best suited intervention to reduce sedentary behaviour with OP in a residential care home	UK & Spain	Co-creation	Reduce sedentary behaviour and enhance PA
Glover [37]	2020	To understand what constitutes healthy ageing in the socioeconomic context	UK	Co-creation	Healthy aging
Guell et al*.* [38]	2018	Develop strategies for motivating each typology	UK	Co-design	PA Typology
Hall et al*.* [39]	2020	To develop an intervention for reducing sedentary behaviour after stroke	UK	Co-production	Sedentary Behaviour
Hatton et al*.* [40]	2020	To develop novel solutions in ageing well with healthy living	UK & Australia	Co-design	Healthy aging
Kirk et al*.* [41]	2021	Design an intervention to increase mobility in OP in hospital	Denmark	Co-design	Increase mobility
Leask et al. [42]	2019	Develop recommendations for redesigning and promoting local leisure services (emphasis on muscle and bone strengthening and balance activities)	UK	Co-creation	Strength & balance
Mansfield et al. [43]	2019	Part of wider community sport project which focused on inactive groups	Australia	Co-production + Participatory community approaches	Understand PA
**Design of Products**					
Borema et al*.* [44]	2017	Demonstrate how value-based design can contribute to the design of mobility aids that address real human needs and lead to high acceptance	Netherlands	Co-creation	A mobile walker
Treadaway and Kenning [45]	2016	To develop new types of sensory e-textiles	UK & Australia	Person-centred co-design	A senor e-textile
**Design of Exergames**					
Da Silva Junior et al*.* [46]	2021	Develop a new exergame to (1) tailor the game mechanics and optimize adherence to and enjoyment of exercise; (2) test the functional capacity, motivation, and adherence to the exergaming program; and (3) compare these scores between those who played alone and those who played with peers	Brazil	Co-design using experience-based design	A bowling game
Eisapour, Cao, and Boger [47]	2020	Investigate whether playing games and interacting with virtual objects in VR could be comparable alternative to (human) therapist-led exercise for PWD	Canada	Participatory Design	A rowing game
**Design of Apps**					
Harrington et al*.* [48]	2018	Co-designed for health and fitness application	USA	Co-design	Health & fitness app
Mansson et al. [24]	2020	Develop a smartphone self-test application for balance and leg strength	Sweden	Co-creation	Balance function app
Sandlund et al. [49]	2018	Explore exercise preferences and motivators for OP in the context of falls prevention from a gender perspective	Sweden	Participatory & Appreciative Action & Reflection	Walking app
Verhoeven [50]	2016	To design a “Happy Walker” APP	Netherlands & Spain	Participatory design	Falls prevention app

*Abbreviations*: *n* number of, *OP* older people, *PA* Physical Activity, *mo* months, *h* hours, *hcps* Health Care Professionals, *ots* Occupational Therapists, *VR* Virtual Reality, *PWD* people with dementia

#### Country

The studies included a total of 9 countries. Four papers were conducted in multiple countries. Three of these spanned across the UK and either Australia or Spain and the final combined paper spanned across the Netherlands and Spain. Of the studies in individual countries, 4 were in the UK alone. Other than 2 studies based in Sweden, the remainder were based in either the USA, the Netherlands, Brazil, Denmark, Australia and Canada.

#### Participant characteristics and contexts

Most included studies reported participant age, all of which were over 55 years. Two papers specifically included participants with Dementia. Contextual settings varied including care homes, pre and post-retirement groups, community groups, people living independently, in semi-independent sheltered accommodation or in long-term care centres.

#### Physical activity related intended outcomes

Twelve of the 16 included studies had different outcomes. The papers with similar outcomes were in the design of interventions or services category. These included two papers with outcomes regarding healthy ageing [37, 40]. Two other papers aimed to reduce Sedentary Behaviour (SB) and increase PA [36, 39]. Of the remaining papers designing interventions or services these included one each with outcomes of PA typology [38], increasing mobility [41], strength and balance [42] and understanding PA [43]. Of the papers designing produces, one outcome included a mobile walker [44] and another a sensor e-textile [45]. Of the papers designing exergames, one outcome was a bowling game [46], and another was a rowing game [47]. Finally, of the papers designing apps, one outcome was a health & fitness app [48], one was a balance function app [24], one was a walking app [49] and one was a falls prevention app [50].

### Self-identified needs for increasing physical activity in older adults

**Fig. 2:**
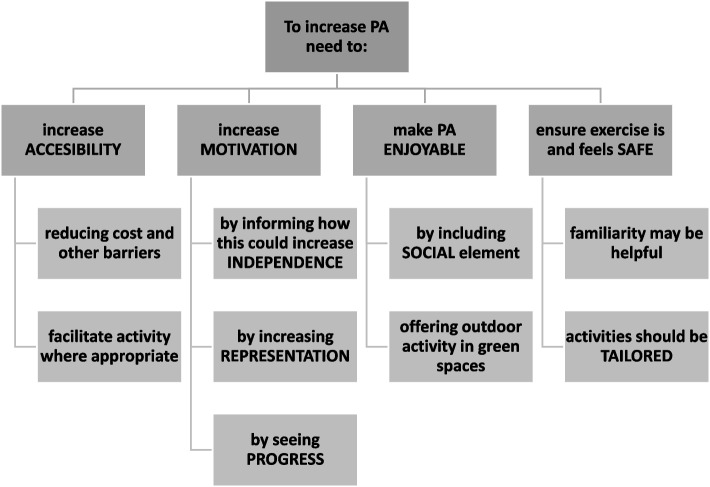
An overview of the main themes and sub-themes identified in the thematic analysis of key results of assets and values identified by Older Adults for increasing Physical Activity

Overall, the main overarching themes identified by older adults to increase activity included accessibility, enjoyment, motivation, and safety (Fig. 2). A summary of these themes and how they map onto the included papers is presented in Table 3.

**Table 3:** Visual representation of the main themes that emerged during analysis of the assets and values identified by Older Adults for increasing Physical Activity

Author (date) [ref]	Main themes (and subthemes)							
	**ACCESIBILITY**			**SAFETY**	**ENJOYMENT**		**MOTIVATION**	
	Bridge barriers	Tailored	Overprotection		Social support	Purposeful	Self-identity	Representation
Gine-Garriga et al. (2019) [36]	**X**	**X**	**X**		**X**	**X**	**X**	**X**
Glover et al. (2019) [37]	**X**			**X**	**X**			**X**
Guell et al. (2018) [38]					**X**	**X**	**X**	
Hall et al. (2020) [39]	**X**	**X**	**X**	**X**	**X**		**X**	**X**
Hatton et al. (2020) [40]					**X**	**X**	**X**	**X**
Kirk et al. (2020) [41]	**X**	**X**	**X**					
Leask et al. (2019) [42]	**X**	**X**	**X**		**X**		**X**	**X**
Mansfield et al. (2019) [43]					**X**	**X**	**X**	
Borema et al. (2016) [44]					**X**	**X**	**X**	
Treadaway and Kenning (2016) [45]	**X**	**X**	**X**		**X**	**X**		
Da silva Junior et al. (2020) [46]				**X**	**X**	**X**		**X**
Eisapour, Cao and Boger (2020) [47]		**X**			**X**	**X**	**X**	**X**
Harrington et al. (2018) [48]		**X**		**X**	**X**		**X**	**X**
Mansson et al. (2020) [24]		**X**		**X**	**X**	**X**		
Sandlund et al. (2018) [49]		**X**			**X**	**X**	**X**	**X**
Verhoeven et al. (2016) [50]	**X**	**X**	**X**	**X**	**X**		**X**	**X**

X indicates where presented themes or sub-theme were identified in analogous paper

**Fig. 3:**
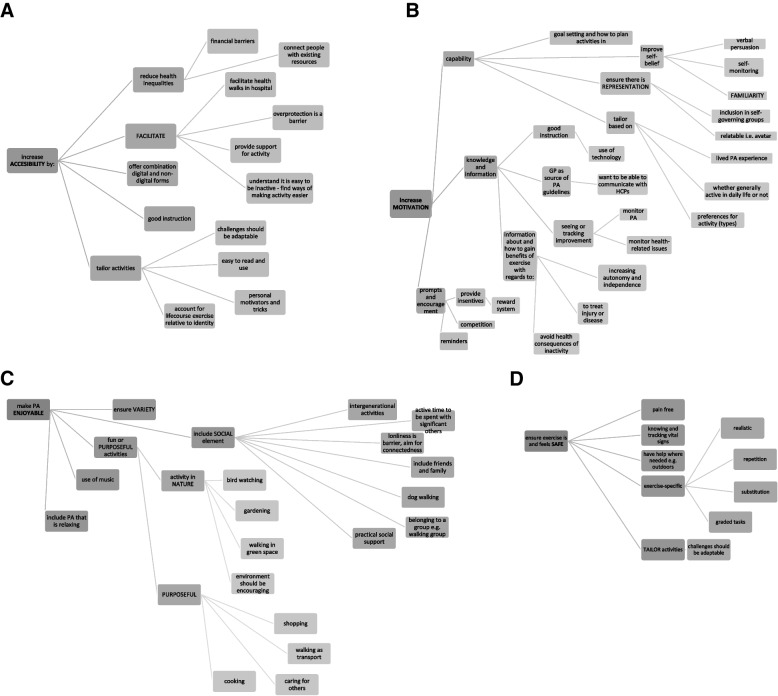
**a** A mind map breakdown of one of the main themes: ACCESSIBILITY. **b** A mind map breakdown of one of the main themes: MOTIVATION. **c** A mind map breakdown of one of the main themes: ENJOYABLE. **d** A mind map breakdown of one of the main themes: SAFETY

A more detailed table of the values and factors thematically analysed from each included papers can be found in additional file 1. A mind map was created in NVivo to make sense of the identified themes. This mind map was split into 4 manageable mind maps to display in Fig. 3(a-d).

#### Theme 1: accessibility

Increasing accessibility of PA was a key theme identified as important by older adults. This included reducing cost and other barriers, in addition to facilitating activity where appropriate, when access or mobility may have impacted activity. Most papers reported increasing accessibility by tailoring the interventions or products being co-created. For example, this included making challenges adaptable, ensuring apps were easy to use and read and accounting for life course experience of PA. Papers also focused on tackling barriers mentioned, including financial barriers, or addressing the barrier of overprotection specifically.

#### Theme 2: motivation

All but 2 papers contributed to the theme of ‘motivation’. This was one of the largest and detailed themes developed when synthesising the literature in this scoping review. The main subthemes within motivation included capability, knowledge and information and use of prompts and encouragement amongst other behaviour change techniques.

#### Theme 3: enjoyable

All but 1 paper discussed social support /activities, and this was often linked to a perception of enjoyment of activity. Activities were described in relation to their effectiveness in making connections with family and friends, making the outcome a meaningful social encounter. Activities were often enjoyed when outdoors or in green spaces (e.g., gardening) or if they were purposeful. Familiar games (e.g., bowling), music and purposeful activities that included interests (e.g., botanical or historical walks) were also discussed as enjoyable.

#### Theme 4: safety

The final main theme was safety. This was a common underlying concern for the environment being a safe space to ensuring activities were pain free and risk-free. For example, by tailoring activities. On occasions, papers referred to over-protection and the ability to assess risk is important for physical activity participation. Older and very old people are known to experience fear of falling, the experience of psychological isolation and cognitive impairment that may impact on safety.

## Discussion

This review was undertaken in preparation for a funded study to identify the priorities for maintaining PA with older adults in sheltered accommodation that would enable a digital platform to be produced (MobMag UKRI Grant G0606-56, forthcoming publications). This scoping review enabled a systematic understanding of the priorities and preferences for continued mobility in older adults. Findings were used to inform the design of the co-production activity.

Overall, studies included in the present review were heterogeneous, with few methodological approaches in common to elicit the values of PA and how to promote PA with older adults. Papers varied in their aims, from intervention design to app development and from their approach to PA, whether the aim was to reduce sedentary behaviour, or to promote PA directly. In line with wider literature, there was no consensus of how to approach this common issue, whether it is better to reduce or promote behaviours [51]. However, a set of interrelating themes was developed to make sense of how to best encourage PA in older adults. Thematic analysis of the literature produced mind maps to illustrate the nuances of the older adult experience of PA and mobility. Themes are mapped to the commentaries in the papers and helped synthesise the critical understanding of older adults in each theme domain, discussed in more detail below.

### Older adults want to be motivated to be active

One of the key findings from the present review was the older adults tended to suggest ways of increasing motivation, to be more active. Open coding of key results from included papers lead to themes of capability and motivation, which suggested alignment with the Capability Opportunity Motivation (COM-B) model of behaviour change [52]. This model focuses on individual behaviour change as a mechanism of increasing PA or reducing sedentary behaviours with motivation being a key facets, along with capability, and opportunity [52]. Physical activity was enhanced when activities were tailored based on familiarity and lived experiences [43], general and daily activity levels [38], in addition to preferred types of activity [43]. However, contextual factors were also important, social networking, environmental factors and organisational factors were clearly influential. Opportunity and ‘access’ issues, whether personal or based on limited opportunities in community, suggests that COM-B overlooks the wider systemic constraint to activity, which also became apparent in the results of the present review. In contrast, the WHO age-friendly cities framework focuses on some of these wider systemic constraints to activity, emphasising 8 interconnected domains of urban life [53]. Highlighted in the following sections, results of the present review also aligned well with these 8 domains, for example identifying the need for social inclusion and participation and outdoor spaces. Therefore, rather than focusing exclusively on individual level behaviour change, it is important to consider interventions at the environmental, community and policy level, which target these domains.

### Independence

Older adults often stated they wanted to maintain their independence. Therefore, knowledge of activities that could promote independence [36, 43, 44], reduce the need for informal care [44, 49] or avoid ill-health related with inactivity [36, 44, 49] were motivating. This included providing information for how to gain benefits of exercise [39, 42, 49] and providing good instruction for activity [39], for example via use of assistive technology [42, 48] that could link with healthcare [39, 40]. Supervising exercise sessions or providing opportunities for activity that promote independence could therefore be helpful for increasing activity in this population. It is important, however, to understand what independence means within a targeted community of older adults, and how they would recommend activities could be implemented. The studies reviewed do not address notions of health literacy and or older adult agency in their own communities and this was mainly because the co-production question related to what PA would be preferred but not how it would be delivered. There are clearly examples of where older adults self-organise and support communities with PA [54] where policy and planning was informed by a model of enabling PA in community [55]. Therefore, a focus on how to support communities with PA could be beneficial going forward.

### Older adults want to enjoy their activities

Older adults identified that when they enjoyed activities, they were more likely to remain active and take part in these activities. Ensuring a variety of options [24, 45, 47], using music [43, 49] or focusing on purposeful activities [38] could make activities enjoyable for older people. Ensuring there is interest in the activities on offer, and that these are enjoyed by older adults could increase intrinsic motivation for PA [56]. Activities in nature were also often discussed, including bird watching [38], gardening [36, 44, 49] or just generally being in green spaces [38, 40]. While increased PA is associated with some of the positive effects of exposure to green spaces [57], other factors, for example links with improved mental health and well-being and reduced stress could explain why older adults identified green spaces as important for enjoyment of PA [58]. Enjoyment, or pleasure, associated with being in green spaces could also be linked with the sensual pleasures of being outdoors, which while not directly identified in the present data, links with other conceptual work exploring pleasure of PA in older age in more depth [59]. This knowledge has important implications when considering PA promotion in older adults. For example, the micro-environment surrounding older adults is important for mental health and cognition [60], which cannot be addressed at an individual level. Green space generally can have profound impacts on older adults’ general health [61]. However, the provision of green space is a wider contextual constraint, influenced by policy decisions. Therefore, solutions to increasing provision of green spaces with the aims of promoting PA in older adults need to consider access and safety of these spaces for older adults [62]. This is a systems-level constraints which requires a multi-disciplinary approach to provide solutions for and successfully increasing PA.

### Social element

Where activities were more enjoyable, including a social element, older adults were more inclined to take part in these activities. Within the theme of enjoyment, a social element to activity emerged as a strong subtheme. Older adults identified the importance of taking part in physical activities with others, including friends and family [38, 44, 46, 48]. Intergenerational activities were also highlighted as important [38, 40], including people from younger generations in activities with them. This finding is particularly significant, as many older adults experience loneliness, resulting in both physical and mental health implications, including risk of depression, worse sleep and/or cardiovascular diseases [63, 64]. Loneliness has been the target of varying social therapeutic interventions, with mixed results [65]. The concept of being able to target loneliness via PA interventions could be an effective use of resources with even greater benefit. The effects of PA on psychosocial outcomes is inconsistent [66]. However, if PA is specifically designed to and addresses a social element, connecting older adults together and including intergenerational activity, this may be an effective way to reduce loneliness and improve psychosocial outcomes, in addition to being more enjoyable for older adults and increasing likelihood they will continue to take part in PA.

### Older adults need PA to be accessible and want to feel safe when being active

Most of the included papers reported accessibility as a key barrier, or a possible facilitator for activity. To increase PA, accessibility needs to be increased, for example by reducing cost and other barriers to activity and by facilitating activity where appropriate. While aiming to increase motivation for activity could be seen as an individual level factor for behaviour change, and behaviour change strategies may be employed, when taken together with the ‘access’ theme, it is important to recognise wider constraints to activity in older adults.

### Reducing barriers

Overprotection was stated as a barrier to activity, for example by Gine-Garriga [36] and Kirk et al*.* [41]. However, Kirk et al*.* [41] further emphasised that to increase activity, older adults need to be well supported. Ensuring PA contexts are inclusive to each person’s abilities and providing support for activity through health care professionals, supervised exercise classes or informally through family and friends could better increase PA. Rather than avoiding activity or being discouraged, older adults can instead work within their capabilities with adequate support. It’s worth noting that overprotection usually appears due to increased risk of falls in older adults [67], and links with ‘safety’ as discussed earlier in the paper. Often, the onus is placed on individuals, on older adults themselves, to try find and access PA that is safe, with PA encouraged at an individual level. However, if the environment was safer and friendlier for use by older adults, then perhaps this would be less of an issue.

Building on the key theme of ‘safety’, older adults wanted to ensure activities were both safe and felt safe, and pain free. Older adults were less inclined to take part in activity if they did not feel safe or if they felt pain. It is estimated that multi-morbidity in older adults in England will continue to increase over time [68]. This includes morbidities such as arthritis and stroke, which could impact on ability to undertake PA [69]. Risk of falls in older adults is also often of concern, especially when PA is not tailored or is not offered in safe environmental contexts [67]. Therefore, it is important that PA offered is safe and acceptable to older people. This is a system issue which needs to be addressed at policy and environmental levels, to preserve independence through ageing and ensuring the benefits of PA continue to outweigh the risks for older adults.

Making PA accessible was also discussed in the context of reducing health inequalities. This included aiming to address financial barriers [42], for example, by connecting older adults with existing resources [37]. Addressing these financial barriers through policy-level interventions may also help reduce health inequalities present in the older population [70]. For PA to be inclusive and to reduce health inequalities, it is important these wider access barriers are addressed when designing PA interventions.

## Strengths and Limitations

One of the main considerations needed when reading and interpreting the present review, is the understanding of how the term ‘co-production’ was used, and how this may be problematic going forward. The present review used the term co-production to encompass varying co approaches, including co-design and co-creation. It is important to note that while these terms were included together, these are not interchangeable terms, and should not be used interchangeably [32, 33]. The present review aimed to synthesise qualitative PA-related results based on these varying, but distinctly different approaches. These were combined in the present review to give enough PA-related qualitative data for which to make meaningful synthesis. However, it is important that PA research acknowledges the distinct differences between each of these co-approaches and does not use these terms interchangeably. Examples of which approach is best to use and how to use these exist, even applied across other PA contexts [71]. Future PA research in older adults should therefore be clear on what type of co-approach is being used and why.

The present review also had several other limitations. Firstly, papers included were heterogeneous, limiting the ability to make strong conclusions. However, the topics and themes examined within each diverse paper were all linked, therefore emerging themes were unlikely impacted by the differing methods employed. Results from different perspectives may have aided a more thorough synthesis of themes, with better understanding of the interrelationships present. Secondly, the present review applied the synthesis of the mind maps to better understand the links between the constructed themes. This synthesis method aided understanding of links between constructed themes, however, could be influenced by the bias of the researchers interpreting and organising themes. Other researchers with different views, experiences and backgrounds may synthesise these themes differently. However, the identified themes and interrelationships identified linked well with wider literature and theory, therefore a level of confidence can be had in these results.

## Conclusions

The present scoping review identified studies using co-production to identify values and factors important for encouraging PA in older adults. When encouraging PA in older adults, it is important to consider the wider contextual and environmental influences on activity. Opportunities for PA need to be accessible and feel safe. Therefore, removing barriers of mobility, access, cost and ensuring PA is tailored and safe are important considerations at not only individual, but also environmental, community and policy levels. In future, interventions aiming to increase PA in older populations should apply co-production approaches to work in partnership with older adults and address wider contextual constraints.

## Supplementary Information

**Additional file 1:** **Table A1.** Key results of values and factors identified by older people for increasing PA extracted for thematic analysis. 

## Data Availability

All data generated or analysed during this study are included in this published article [and its supplementary information files].

## Abbreviations

PA: Physical activity
WHO: World Health Organisation
UK: United Kingdom
USA: United States of America
