# Supplementary material for: The key values and factors identified by older adults to promote physical activity and reduce sedentary behaviour using co-production approaches: a scoping review
Source: BMC Geriatr. 2023 Jun 16;23:371. doi: 10.1186/s12877-023-04005-x (PMC10276377; doi:10.1186/s12877-023-04005-x)
Supplement: Supplementary file 1 — Additional file 1: Table A1. Key results of values and factors identified by older people for increasing PA extracted for thematic analysis. [file 12877_2023_4005_MOESM1_ESM.docx]

## Additional file 1

Summary of key results of values and factors identified by Older Adults for increasing Physical Activity extracted for thematic analysis’.

Table A1. Key results of values and factors identified by Older People for increasing PA extracted for thematic analysis

| **Paper** | **Assets and values identified by Older People for increasing PA** |
| --- | --- |
|  | **Design of Intervention and Services** |
| Gine-Garriga *et al*. (2019) | 1) PA is for health and happiness  2) PA is for everyone  3) Overprotection is a barrier  4) Inactivity is in the walls  5) Longing for autonomy  6) Wanting an improved wellbeing  7) The influence of significant others, incorporating a 5 minute walk into time spent together  8) Prompts and encouragement, where more sedentary work this around the end of a game or chapter  9) Incorporate the enjoyment found from gardening, shopping, cooking, and household cleaning  10) Exercise through the life course relative to identity should play a role in establishing a new interest  11) Open up to the neighbourhood, invite younger people in  12) Go out to exercise, walk to get fish and chips  13) Affirming individual identity is important as a motivator |
| Glover *et al*. (2019) | 1) Social Connectedness is key, loneliness is a barrier  2) Being safe and pain free is more important than exercise  Glover’s project aligned healthy ageing with ‘exercise’ rather than using an alternative expression such as PA, this may have influenced what the older people said about feeling pain free as being” far more important”  3) Rather than developing new interventions, connect people with existing resources and provide a human ‘bridge’ to address barriers to accessing these  4) Create self-governing groups including older people  5) Older people can be recruited to build networks |
| Guell *et al*. (2018) | Three types of OPs-  1) Exercisers - who have engaged in sport and exercise throughout the life  2) Out-and-abouters have engaged in activities socially through pursuing interests  3) People who prefer sedentary/ solitary activities  PA should align with the types preferences.  1) Older people (often those who were once manual workers) desire *purposefu*l activities, historical or botanical walks, bird watching, to see formal gardens, dog walking.  2) Older people prefer activities in the *social world*, and that include friends and family. Dog walking was a popular idea and sharing or borrowing a dog with another a solution to this.  3) Older people prefer *intergenerational* activities to age specific programmes, one idea arising from this was younger people teaching older people how to use fitness apps to track their activities  4) Sometimes those who were solitary still enjoyed activity that included social responsibility such as caring for other family members, grand children for example |
| Hall *et al*. (2020) | 1) Good goals and planning around activities,  2) Self-monitoring of behaviours  3) Practical social support, good instruction  4) Information about health consequences  5) Information about others behaviour to enhance feelings of approval  6) Graded tasks with repetition and substitution, a comparison of outcomes and verbal persuasion to enhance self belief. |
| Hatton *et al*. (2020) | 1) The experience of nature. Interactions with green and blue spaces, community walking maps, social walking  2) Use of media for sensitisation and virtual reality were useful and motivational  3) Interactions with other and especially between younger and older adults participants, are beneficial for physical and social wellbeing. |
| Kirk *et al*. (2020) | 1)Older people in hospital should not be forgotten and should be facilitated to go on Health walks.  2)Older people should not be over protected and if they want to get up should be supported to do so |
| Leask *et al*. (2019) | 1) Knowledge of the PA guidelines for older people can encourage engagement and should be promoted at GP surgeries and pharmacies.  2) Voluntary ambassadors could raise awareness and engage individuals to be more active.  3) Reducing financial barriers to PA participation is important  4) Health inequalities could affect engagement  5) Available activity opportunities should be available in both digital and non-digital formats  6) Incentivisation can be used for those who achieve particular activity levels as a mechanism to improve maintenance |
| Mansfield *et al*. (2019) | 1) Lived experience influences PA preferences  2) Embodying aging personal fulfilment and good health – to be ““fulfilled,” “alive,” and “productive”  3) Corporeal pleasures of older sporting bodies – fun activities (skittles, indoor curling, table tennis, and swimming) include music from the 40, 50, and 60s. |

|  | **Design of Products** |
| --- | --- |
| Borema *et al.* (2016) | 1) Social interaction, family and doing things with others  2) Independence  3) Relaxation  4) Expanding life space/ social world  4) Killing time  5) Good physical condition  6) Not being a burden /reducing the need for informal care  7) Nostalgia /traditions  8) Peace of mind  Ways to keep active broad definitions explored:  1) Doing groceries  2) Hobbies  3) Riding a bike or driving a car |
| Treadaway and Kenning (2016) | 1) Differing textile incorporating textures, smells, sounds, shapes, weights and colours can stimulate PWD to move and conduct upper body movements safely while seated.  2) We need to “bring the world to the person”  3) Pleasure, fun and playfulness are important ways of keeping people with dementia in the moment and enhancing positive emotion. |

|  | **Design of Exergames** |
| --- | --- |
| Da silva Junior *et al.* (2020) | 1) Games should be familiar  2) Contain a social element, something reminiscent of family and friends  3) Have realistic features (similar to a conventional bowling game)  4) Feature an avatar that looks like me, ie age and gender appropriate.  5) Should contain updating / new features  6) Feature competition |
| Eisapour, Cao and Boger (2020) | 1) Exercise should be relaxing - “I love rowing because it was relaxing”  2) The environment should be engaging – “I love looking at a blue sky”  3) Seeing improvement is enjoyable and rewarding - “It was a longer class today, rowing, ... I could [keep] going”  4) The level of challenge should be adaptable, - for some it was too easy for others too difficult.  5) Variety enhances interest - “Too much repetition, more motion needed, everything was the same.” |

|  | **Design of Applications** |
| --- | --- |
| Harrington *et al.* (2018) | 1) An APP that assess and flexibility as a motivator.  2) Knowing other health vital signs such as blood pressure, heart rate.  3) To be able to communicate with health care professionals.  4) A feature that assists in planning a schedule to fit the week.  5) Calorie goal  7) To be able to track improved health and fitness.  8) A reward system.  9) Connection to others, friends using the same APP. Enhance connections.  10) Competition element. |
| Mansson *et al.* (2020) | 1) Would like the benefits of being able to compare their performance to a normal value without competition  2) Would like a Balance App to organise information logically and simply and not be boring  3) Would like to monitor PA or health related issues.  4) Would like to feel safe. |
| Sandlund *et al.* (2018) | Motivators to starting PA –  1) Preserving health (for men this is fitness, for women this is maintaining ability to carry out responsibilities)  2) Treating injury or disease  3) Encouragement by clinicians or relatives  4) Good information on benefits of exercises in question  5) Exercises in everyday activities that incorporate secondary goals such as gardening. cleaning the home, or going out for a walk with a clear purpose.  6) Feeling good afterwards.  7) Use of music or rhythm  8) Belonging in a group, eg walking with poles group  9) Exercise should be joyful.  10) Develop personal tricks – companionship, routines, challenges, dogs and nature. |
| Verhoeven *et al.* (2016) | 1) PA should be activities close to home  2) Should be safe  3) Should foster social contact  4) Apps should be tailored to the individual  5) Apps should be interfaced to suit older people with large fonts and clear icons and cues.  6) Should feature reminders for appointments and activities  7) Have a function to suggest activities,  8) Contain a checklist before going outside  9) Assist in the orderly arrangement of things and outside activities,  10) Include reminders of what is needed for the activity, helping with navigation to improve orientation while moving, coming home in the dark |
